# Analysis of the Neuron Dynamics in Thalamic Reticular Nucleus by a Reduced Model

**DOI:** 10.3389/fncom.2021.764153

**Published:** 2021-11-16

**Authors:** Chaoming Wang, Shangyang Li, Si Wu

**Affiliations:** ^1^School of Psychology and Cognitive Sciences, Peking-Tsinghua Center for Life Sciences, IDG/McGovern Institute for Brain Research, Academy for Advanced Interdisciplinary Studies, Peking University, Beijing, China; ^2^Institute of Artificial Intelligence, Hefei Comprehensive National Science Center, Hefei, China; ^3^Chinese Institute for BrainResearch, Beijing, China

**Keywords:** thalamic reticular nucleus, neuron model, bursting, tonic spiking, bifurcation analysis, phase plane analysis

## Abstract

Strategically located between the thalamus and the cortex, the inhibitory thalamic reticular nucleus (TRN) is a hub to regulate selective attention during wakefulness and control the thalamic and cortical oscillations during sleep. A salient feature of TRN neurons contributing to these functions is their characteristic firing patterns, ranging in a continuum from tonic spiking to bursting spiking. However, the dynamical mechanism under these firing behaviors is not well understood. In this study, by applying a reduction method to a full conductance-based neuron model, we construct a reduced three-variable model to investigate the dynamics of TRN neurons. We show that the reduced model can effectively reproduce the spiking patterns of TRN neurons as observed *in vivo* and *in vitro* experiments, and meanwhile allow us to perform bifurcation analysis of the spiking dynamics. Specifically, we demonstrate that the rebound bursting of a TRN neuron is a type of “fold/homo-clinic” bifurcation, and the tonic spiking is the fold cycle bifurcation. Further one-parameter bifurcation analysis reveals that the transition between these discharge patterns can be controlled by the external current. We expect that this reduced neuron model will help us to further study the complicated dynamics and functions of the TRN network.

## 1. Introduction

The thalamic reticular nucleus (TRN) is a brain area containing a large population of GABAergic neurons (Sherman and Guillery, 2001; Pinault, 2004; Halassa and Acsády, 2016; Crabtree, 2018). It receives glutamatergic projections from the thalamocortical and corticothalamic neurons (Sherman and Guillery, 2001; Pinault, 2004; Crabtree, 2018), and inhibitory and modulatory inputs from many subcortical regions (Jourdain et al., 1989; Govindaiah et al., 2010; Beierlein, 2014; Herrera et al., 2016; Nakajima et al., 2019). However, all its efferents are targeted on cells in the thalamic nuclei (Sherman and Guillery, 2001; Pinault, 2004; Crabtree, 2018), and nearly every part of the thalamus receives inhibition from the TRN (Swanson et al., 2019). Located in such a strategical position, TRN has long been hypothesized to play an important role in the regulation of information flow transferred through the thalamus. In an influential theoretical proposal, Francis Crick suggested that “if the thalamus is the gateway to the cortex, the reticular complex might be described as the guardian of the gateway” (Crick, 1984). This hypothesis of the importance of TRN on selective attention has been confirmed by recent studies on rodents, monkeys, and humans (McAlonan et al., 2006, 2008; Halassa et al., 2014; Ahrens et al., 2015; Wimmer et al., 2015; Wells et al., 2016; Nakajima et al., 2019). Moreover, a growing body of experiments starts to draw the conclusion that TRN is also a center for the control of the brain oscillation during sleep. The pioneering work of Steriade et al. (1985, 1987) found that, in cats *in vivo*, the TRN-disconnected thalamic nuclei lose their ability to generate spindle oscillations, while the isolated TRN deprived of inputs from the cortex and thalamus can itself generate spindle rhythms, demonstrating TRN is the spindle pacemaker. Moreover, optogenetic studies have shown that activation pulses on TRN neurons can evoke spindle oscillations in the connected thalamic or cortical areas (Halassa et al., 2011; Barthó et al., 2014; Clemente-Perez et al., 2017). Another study in awake mice found that local activation of TRN can rapidly induce delta oscillation in the corresponding region of the cortex area, along with a decrease in arousal state (Lewis et al., 2015). Putting together, these experimental results suggest that TRN has strong functional controls on attentional filtering and oscillation regulation in the brain.

A key ingredient of TRN neurons to exert their functions in the brain is their rich firing patterns, including bursts (low-threshold Ca^2+^ potentials) when neurons are hyperpolarized (e.g., during sleep) (Steriade et al., 1986; Huguenard and Prince, 1992; Bal and McCormick, 1993; Llinás and Steriade, 2006), tonic spikes when neurons are depolarized (e.g., during the wakefulness or attention state) (Steriade et al., 1986, 1993; Herd et al., 2013; Rovó et al., 2014; Lewis et al., 2015), and firing patterns between them (Pinault, 2004; Halassa and Acsády, 2016). On the one hand, the intrinsic bursting in TRN neurons is important for the regulation of brain oscillations. Barthó et al. (2014) demonstrated that the number of spikes in each TRN burst predicts the progression of a spindle event. Moreover, genetic deletion of Ca^2+^ channels in TRN neurons in mice abolished low-threshold Ca^2+^ potentials and bursts, which further suppressed spindle rhythms while enhanced delta oscillations during sleep (Pellegrini et al., 2016; Fernandez et al., 2018; Vantomme et al., 2019; Li et al., 2020). On the other hand, TRN tonic spikes are prevalent during the wakefulness or attentional state (Pinault, 2004; McAlonan et al., 2006, 2008). Such tonic inhibition permits information transfer from the thalamus during an attentional focus (McAlonan et al., 2006, 2008; Coulon and Landisman, 2017), and allows fine-grained inhibitory control on the thalamic targets (McAlonan et al., 2008; Wimmer et al., 2015; Halassa and Acsády, 2016).

To reproduce these characteristic firing patterns, detailed conductance-based neuron models were proposed for TRN neurons (Destexhe et al., 1996; Bazhenov et al., 1998, 2002; Vijayan and Kopell, 2012; Fan et al., 2017). However, in these full models, too many voltage-dependent conductance channels and dynamical parameters are involved, which makes it hard to analyze the neuronal dynamics clearly and, hence, prevents us from understanding the inner mechanism underlying the rich dynamics of TRN neurons. Furthermore, it has been documented that distributed TRN cells in different regions usually exhibit different spiking properties (Brunton and Charpak, 1997; Lee et al., 2007; Clemente-Perez et al., 2017; Li et al., 2020; Martinez-Garcia et al., 2020). A full neuron model with too many parameters makes it hard to identify the key elements that contribute to this intrinsic heterogeneity in firing patterns. Without understanding the dynamics of single TRN neurons clearly, it will be difficult for us to understand the much more complicated dynamics and functions of the TRN network.

In this study, motivated by the foregoing shortcomings of a full neuron model, we present a reduced three-variable model for TRN neurons by adopting a reduction method proposed by Kepler et al. (1992). The main idea of this reduction algorithm is to group different variables operating in a similar time scale into a single representative variable. The reduced model is an approximation of the original full model but preserves its key dynamical features. The reduced model also keeps the original biological meanings of the corresponding full model, as the individual channel currents can be recovered from the reduced variables. The key advantage of the reduced TRN neuron model is that we can perform theoretical analysis on its dynamics. Utilizing the reduced model, we carry out an analysis to elucidate the bifurcation mechanisms under two characteristic discharge patterns of TRN neurons, i.e., the rebound bursting and tonic spiking. We show that the rebound bursting is a type of “fold/homo-clinic” bifurcation and the tonic spiking is the fold cycle bifurcation. We also carry out a one-parameter bifurcation analysis and find that transition between these two firing patterns can be achieved by varying the external input. We hope that this study will pave the way for us to study the much more complicated dynamics of the TRN network in the future study.

## 2. A Full Model of TRN Neurons

The full mathematical model of TRN neurons we consider was proposed by Bazhenov et al. (1998, 2002), which is described as a single-compartment Hodgkin-Huxley schema, with a set of active channels sufficient to produce the typical firing patterns seen in TRN neurons. Similarly to other dynamical systems that describe isopotential excitable neural membranes, this model could be written as this form:


(1) $$\begin{array}{l} C\frac{\text{d}V}{\text{d}t}+I\left(V,\left\{x_{i}\right\}\right)=\frac{10^{-3}I_{syn}\left(t\right)}{A}, \end{array}$$

where *V* is the membrane potential, *C* = 1 μF/cm^2^ is the membrane capacitance, *I*(*V*, {*x*_*i*_}) is a term expressed as a function of *V* and the gating variables *x*_*i*_ describing the ion currents, and *I*_*syn*_ is the received synaptic current which is normalized by the membrane area *A* = 1.43 × 10^−4^cm^2^ (Bazhenov et al., 2002). Specifically, this model contains three active ionic currents, which are fast sodium current *I*_*Na*_ and a delayed rectifier *I*_*K*_ for spiking generation, and a low-threshold Ca^2+^ current *I*_*T*_ for rebound bursting, and two leaky currents, which are a membrane leakage current *I*_*L*_ and a potassium leaky current *I*_*KL*_ controlled by neuromodulators like acetylcholine and norepinephrine (McCormick, 1992). The ion current term is written as,


(2) $$\begin{array}{l} I\left(V,\left\{x_{i}\right\}\right)=I_{L}+I_{KL}+I_{Na}+I_{K}+I_{T}, \end{array}$$

where the leakage current is modeled as *I*_*L*_ = *g*_*L*_(*V*−*E*_*L*_), with the leakage channel conductance $g_{L}=0.06\text{ mS}/\text{c}{\text{m}}^{2}$ and the leakage reversal potential *E*_*L*_ = −70 mV, and the potassium leaky current is modeled as *I*_*KL*_ = *g*_*KL*_(*V* − *E*_*KL*_), with the reversal potential *E*_*KL*_ = −100 mV. The last three items are voltage-dependent ionic currents which are expressed in a unified form as,


(3) $$\begin{array}{l} I_{i}=g_{i}M^{j}N^{k}\left(V-E_{i}\right),\;i\in \left\{Na,K,T\right\} \end{array}$$

where *g*_*i*_ is the maximal conductance, *M* is the activation gating variable, *N* is the inactivation gating variable, *j* and *k* are the numbers of the gating variables, and *E*_*i*_ is the reversal potential. Specifically, $I_{Na}=g_{Na}m^{3}h\left(V-E_{Na}\right)$, $I_{K}=g_{K}n^{4}\left(V-E_{K}\right)$, and $I_{T}=g_{T}p^{2}q\left(V-E_{T}\right)$, with the maximal conductance $g_{Na}=100\text{ mS}/\text{c}{\text{m}}^{2}$, $g_{K}=10\text{ mS}/\text{c}{\text{m}}^{2}$, and the reversal potentials *E*_*Na*_ = 50 mV, *E*_*K*_ = −100 mV, and *E*_*T*_ = 120 mV. The dynamics of gating variables are expressed in the below unified form,


(4) $$\begin{array}{l} \frac{d\theta }{dt}=\phi_{\theta }\frac{\theta_{\infty }\left(V\right)-\theta }{\tau_{\theta }\left(V\right)},\;\theta \in \left\{m,h,n,p,q\right\}, \end{array}$$

where θ_∞_ is the voltage-dependent steady state, τ_θ_ is the voltage-dependent time constant, and ϕ_θ_ is a constant. For a gating variable θ ∈ {*m, h, n*}, θ_∞_ and τ_θ_ are expressed by the voltage-dependent state transition variables α and β, i.e.,


(5) $$\begin{array}{l} \theta_{\infty }\left(V\right)=\frac{\alpha_{\theta }\left(V\right)}{\alpha_{\theta }\left(V\right)+\beta_{\theta }\left(V\right)},\;\tau_{\theta }=\frac{1}{\alpha_{\theta }\left(V\right)+\beta_{\theta }\left(V\right)}. \end{array}$$

The equations and values of the above variables are listed in Table 1.

**Table 1:** Kinetics of gating variables.

**θ**	**ϕ_θ_**	**α_θ_(*V*) or θ_∞_(*V*)**	**β_θ_(*V*) or τ_θ_(*V*)**	**Reference**
*m*	1	$\alpha_{m}=\frac{0.32\left(13.-V+V_{th}^{Na}\right)}{\exp\left(\left(13.-V+V_{th}^{Na}\right)/4\right)-1}$	$\beta_{m}=\frac{0.28\left(V-40.-V_{th}^{Na}\right)}{\exp\left(\left(V-40.-V_{th}^{Na}\right)/5\right)-1}$	①
*h*	1	$\alpha_{h}=0.128\exp\left(\frac{17-V+V_{th}^{Na}}{18}\right)$	$\beta_{h}=\frac{4}{1+\exp\left(\left(40-V+V_{th}^{Na}\right)/5\right)}$	①
*n*	1	$\alpha_{n}=\frac{0.032\left(15-V+V_{th}^{K}\right)}{\exp\left(\left(15-V+V_{th}^{K}\right)/5\right)-1}$	$\beta_{n}=0.5\exp\left(\frac{10-V+V_{th}^{K}}{10}\right)$	①
*p*	6.9	$p_{\infty }=\frac{1}{1+\exp\left(\frac{-52-V+V_{th}^{T}}{7.4}\right)}$	$\tau_{p}=3+\frac{1}{\exp\left(\frac{V+27-V_{th}^{T}}{10}\right)+\exp\left(\frac{-V-102+V_{th}^{T}}{15}\right)}$	②
*q*	3.7	$q_{\infty }=\frac{1}{1+\exp\left(\frac{80+V-V_{th}^{T}}{5}\right)}$	$\tau_{q}=85+\frac{1}{\exp\left(\frac{V+48-V_{th}^{T}}{4}\right)+\exp\left(\frac{-V-407+V_{th}^{T}}{50}\right)}$	②

*The spike adjusting threshold $V_{th}^{Na}=V_{th}^{K}=-55\text{ mV}$ and the calcium channel threshold $V_{th}^{T}=-3mV$*. *The references ① and ② denote Bazhenov et al. (2002) and Huguenard and Prince (1992), respectively*.

## 3. Reduction of the Full Model

As described above, the full model has six dynamical variables (*V*, *m*, *h*, *n*, *p*, and *q*), making it hard to analyze the inner mechanism and difficult to accommodate TRN neurons with different firing patterns. In this study, by adopting a reduction method proposed for the conductance-based neuron model (Kepler et al., 1992), we present a reduced model with a minimal set of variables to capture the behaviors of the original full model. The overall reduction procedure can be summarized into the following four steps.

### 3.1. Step 1: Converting Gating Variables Into Equivalent Potentials

In the original expressions, variables are not dimensionally equivalent. To combine different variables for reduction, making them dimensionally equivalent is necessary. Membrane potential provides a connection between these variables, so we consider that all gating variables are converted into equivalent potentials *v*_θ_, in term of that in the voltage-clamp recording, equilibrium voltage will gives rise to the value θ, i.e., θ = θ_∞_(*v*_θ_) or equivalently, $v_{\theta }=\theta_{\infty }^{-1}\left(\theta \right)$.

Using the equivalent potentials, the original full model Equation (1) can be re-expressed as,


(6) $$\begin{array}{l} C\frac{\text{d}V}{\text{d}t}+F\left(V,\left\{v_{\theta }\right\}\right)=\frac{10^{-3}I_{syn}\left(t\right)}{A}, \end{array}$$

where


(7) $$\begin{array}{l} F\left(V,\left\{v_{\theta }\right\}\right)=I\left(V,\left\{\theta_{\infty }\left(v_{\theta }\right)\right\}\right)\\ \;=g_{Na}m_{\infty }^{3}\left(v_{m}\right)h_{\infty }\left(v_{h}\right)\\ \;+g_{T}p_{\infty }^{2}\left(v_{p}\right)q_{\infty }\left(v_{q}\right)\left(V-E_{T}\right)\\ \;+g_{K}n_{\infty }^{4}\left(v_{n}\right)\left(V-E_{K}\right)+I_{L}+I_{KL}. \end{array}$$

From Equation (5), we also obtain


(8) $$\begin{array}{l} \frac{dv_{\theta }}{dt}=\phi_{\theta }\frac{\theta_{\infty }\left(V\right)-\theta_{\infty }\left(v_{\theta }\right)}{\tau_{\theta }\left(V\right)\theta_{\infty }^{\prime }\left(v_{\theta }\right)}=f_{\theta }\left(V,v_{\theta }\right),\;\theta \in \left\{m,h,n,p,q\right\}, \end{array}$$

where $\theta_{\infty }^{\prime }$ denotes the derivative of θ_∞_ with respect to *v*_θ_.

### 3.2. Step 2: Grouping Variables at a Similar Time Scale

**Figure 1:**
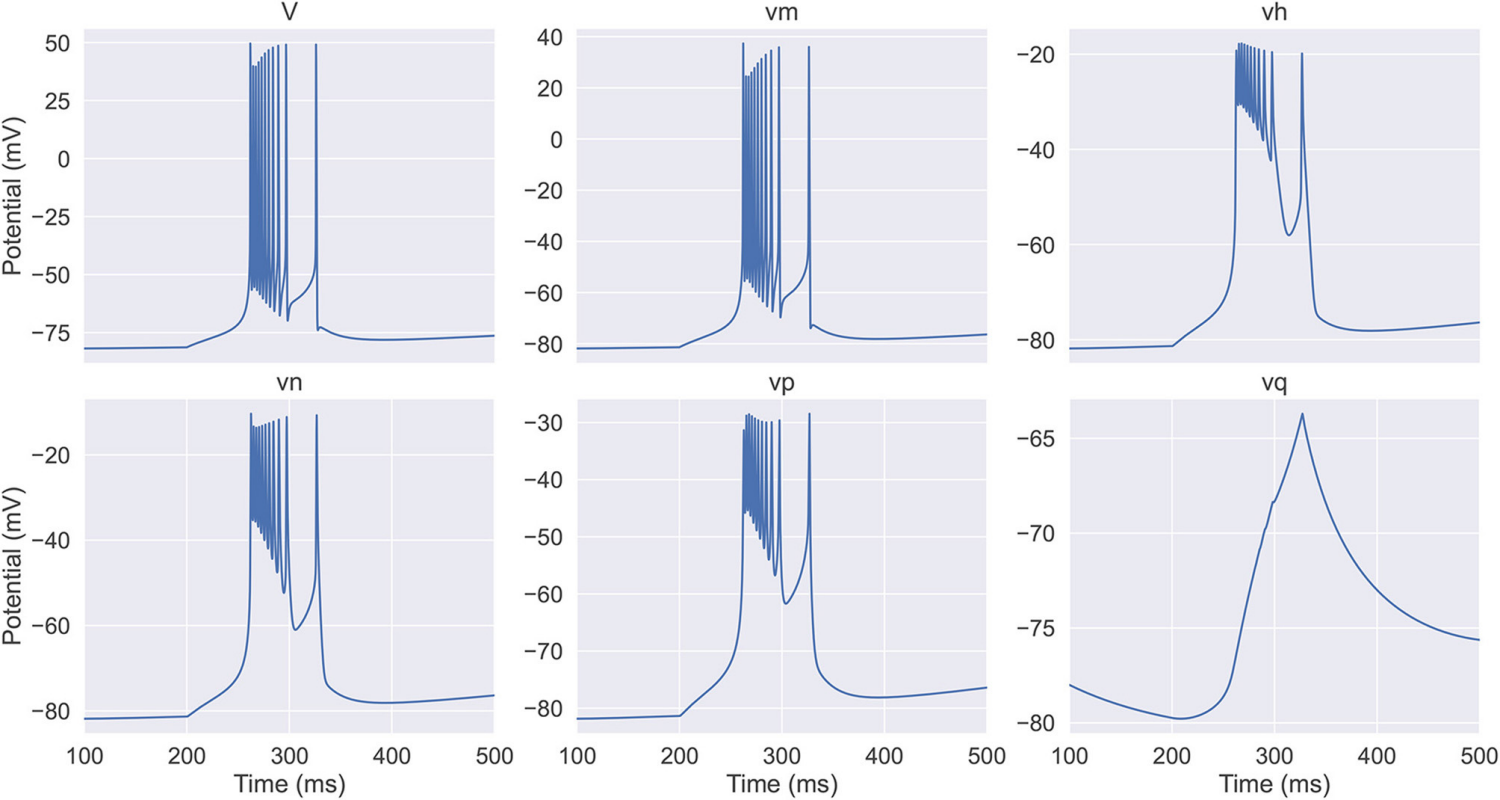
The evolution of the membrane potential and the equivalent potentials in the full thalamic reticular nucleus (TRN) neuron model. The model (Equations 6–8) receives an inhibitory current with the size of −0.03 mS/cm^2^ during the first 200 ms. In the next 400 ms, the model exhibits a rebound bursting. *V* and *v*_*m*_ have the fastest time scale and exhibit the largest variations; *v*_*h*_, *v*_*n*_, and *v*_*p*_ response at a slower time scale and display intermediate amplitude variations; *v*_*q*_ has the slowest time constant and shows the modest variation. The neuronal parameters used are *E*_*L*_ = –77 mV, $g_{KL}=0.00793\text{ mS}/\text{c}{\text{m}}^{2}$, and $g_{T}=2\text{ mS}/\text{c}{\text{m}}^{2}$.

By simulation, we obtain the dynamics of equivalent potentials in the full TRN neuron model, which are displayed in Figure 1. We see that they can be categorized into three classes. The first category includes *V* and *v*_*m*_, which have the largest variations. The second category includes *v*_*h*_, *v*_*n*_, and *v*_*p*_, which have an intermediate amplitude of variation and change on a slower time scale compared to the first category. Note that *v*_*h*_ and *v*_*n*_ are anti-synergistic to *v*_*p*_, in terms that the increases in *v*_*h*_ and *v*_*n*_ hyper-polarize the membrane potential while the increase in *v*_*p*_ depolarizes the membrane potential. The third category only includes *v*_*q*_, which has the smallest amplitude of variation and changes at the slowest speed. These properties suggest that the dynamics of a TRN neuron in the six-dimensional space can be effectively reduced to a three-dimensional manifold.

To locate this manifold, we group those equivalent potentials behaving similarly into a single representative variable as a weighted average of their values, which are written as,


(9) $$\begin{array}{l} x=\rho_{V}V+\rho_{m}v_{m}, \end{array}$$

(10) $$\begin{array}{l} y=\rho_{h}v_{h}+\rho_{n}v_{n}+\rho_{p}v_{p}, \end{array}$$

(11) $$\begin{array}{l} z=v_{q}, \end{array}$$

where all the coefficients {ρ_*i*_}, for *i* ∈ {*V, m, h, n, p, q*}, are non-negative, and they satisfy ρ_*V*_ + ρ_*m*_ = 1 and ρ_*h*_ + ρ_*n*_ + ρ_*p*_ = 1.

### 3.3. Step 3: Determining the New Variables

We fix the coefficients for the new variables in Equations (9–11) under the condition that the dynamics of the membrane potential of the reduced model can approximate that of the full model as accurately as possible.

From Equation (9), we have


(12) $$\begin{array}{l} \frac{dx}{dt}=\rho_{V}\frac{dV}{dt}+\rho_{m}\frac{dv_{m}}{dt}. \end{array}$$

Substituting Equations (6–8) into Equation (12) and approximating the dynamics in the first-order Taylor expansion, we have,


(13) $$\begin{array}{l} C\frac{\text{d}x}{\text{d}t}=\rho_{V}\frac{10^{-3}I_{syn}(t)}{A}\\ \;-[\rho_{V}F(x,y,z)+\delta_{h}\rho_{V}\frac{\partial F}{\partial v_{h}}+\delta_{n}\rho_{V}\frac{\partial F}{\partial v_{n}}+\delta_{p}\rho_{V}\frac{\partial F}{\partial v_{p}}\\ \;+\delta_{V}\left(\rho_{V}\frac{\partial F}{\partial V}-\frac{C\phi_{m}\rho_{m}}{\tau_{m}}\right)\\ \;+\delta_{m}\left(\rho_{V}\frac{\partial F}{\partial v_{m}}+\frac{C\phi_{m}\rho_{m}}{\tau_{m}}\right)+O(\delta^{2})], \end{array}$$

where δ_*j*_ = *x*−*j*, for *j* ∈ {*V, v*_*m*_}, and δ_*l*_ = *y*−*l*, for *l* ∈ {*v*_*h*_, *v*_*n*_, *v*_*p*_}. It is straightforward to check that ρ_*h*_δ_*v*_*h*__ + ρ_*n*_δ_*v*_*n*__ + ρ_*p*_δ_*v*_*p*__ = 0, and ρ_*V*_δ_*V*_ + ρ_*m*_δ_*v*_*m*__ = 0. To get the above result, we have used the conditions that *f*_*m*_(*x, x*) = 0 and ∂*f*_*m*_(*x, x*)/∂*V* = −∂*f*_*m*_(*x, x*)/∂*v*_*m*_ = ϕ_θ_/τ_*m*_.

We determine the coefficients {ρ_*i*_} by requiring that the discrepancy between the reduced and the full models (i.e., the first-order Taylor expansion in Equation 13) be as small as possible. This is formulated as an optimization problem. Specifically, for determining the coefficients ρ_*h*_, ρ_*n*_, ρ_*p*_, ρ_*m*_, *andρ*_*V*_, the optimization problem is formulated as follows,


(14) $$\begin{array}{l} \;\min|\delta_{v_{h}}\rho_{h}\frac{\partial F}{\partial v_{h}}+\delta_{v_{n}}\rho_{n}\frac{\partial F}{\partial v_{n}}+\delta_{v_{p}}\rho_{v_{p}}\frac{\partial F}{\partial v_{p}}\\ \;+\delta_{V}\left(\rho_{V}\frac{\partial F}{\partial V}-\frac{C\phi_{m}\rho_{m}}{\tau_{m}}\right)\\ \;+\delta_{v_{m}}\left(\rho_{V}\frac{\partial F}{\partial v_{m}}+\frac{C\phi_{m}\rho_{m}}{\tau_{m}}\right)|\\ \begin{array}{cccc} \text{s}.\text{t}.\; & \rho_{h}\delta_{v_{h}}+\rho_{n}\delta_{v_{n}}+\rho_{p}\delta_{v_{p}}=0, & \; & \rho_{h},\rho_{n},\rho_{p}\geq 0,\\ & \rho_{V}\delta_{V}+\rho_{m}\delta_{v_{m}}=0, & \; & \rho_{m},\rho_{V}\geq 0, \end{array} \end{array}$$

where the symbol |·| denotes the absolute value. This optimization problem can be solved by using the Lagrangian method (as shown in details in Supplementary Material), which gives,


(15) $$\begin{array}{l} \rho_{p}=k, \end{array}$$

(16) $$\begin{array}{l} \rho_{h}=\left(1-k\right)\frac{\partial F}{\partial v_{h}}/\left(\frac{\partial F}{\partial v_{h}}+\frac{\partial F}{\partial v_{n}}\right), \end{array}$$

(17) $$\begin{array}{l} \rho_{n}=\left(1-k\right)\frac{\partial F}{\partial v_{n}}/\left(\frac{\partial F}{\partial v_{h}}+\frac{\partial F}{\partial v_{n}}\right), \end{array}$$

(18) $$\begin{array}{l} \rho_{V}=\frac{-\left(C\frac{\phi_{m}}{\tau_{m}}+\frac{\partial F}{\partial V}\right)+\sqrt{{\left(C\frac{\phi_{m}}{\tau_{m}}+\frac{\partial F}{\partial V}\right)}^{2}-4C\frac{\phi_{m}}{\tau_{m}}\left(\frac{\partial F}{\partial V}+\frac{\partial F}{\partial v_{m}}\right)}}{-2\left(\frac{\partial F}{\partial V}+\frac{\partial F}{\partial v_{m}}\right)}, \end{array}$$

(19) $$\begin{array}{l} \rho_{m}=1-\rho_{V}, \end{array}$$

where 0 < *k* < 1 is an undetermined constant, which we normally set *k* to be 0.01.

### 3.4. Step 4: Formulating the Reduced Model

After fixing the new variables, we can write down their approximated dynamics. Specifically, for the variable *x* (corresponding to the membrane potential), its dynamics is written as,


(20) $$\begin{array}{l} \frac{C}{\rho_{V}}\frac{dx}{dt}=-g_{Na}m_{\infty }^{3}\left(x\right)h_{\infty }\left(y\right)\left(x-E_{Na}\right)-g_{K}n_{\infty }^{4}\left(y\right)\left(x-E_{K}\right)\\ \;-g_{T}p_{\infty }^{2}\left(y\right)q_{\infty }\left(z\right)\left(x-E_{T}\right)-I_{L}-I_{KL}+\frac{10^{-3}}{A}I_{syn}\left(t\right). \end{array}$$

In this study, we have ignored the discrepancy term in Equation (13) from the full model. Note that *x* has become an alternative variable of membrane potential *V* (in the following section we refer to *V* as *x*).

For the variable *y*, based on Equation (10), we get its dynamics,


(21) $$\begin{array}{l} \frac{dy}{dt}=\rho_{h}\frac{dv_{h}}{dt}+\rho_{n}\frac{dv_{n}}{dt}+\rho_{p}\frac{dv_{p}}{dt},\\ \;=\rho_{h}\phi_{h}\frac{h_{\infty }\left(x\right)-h_{\infty }\left(y\right)}{\tau_{h}\left(x\right)h_{\infty }^{\prime }\left(y\right)}+\rho_{n}\phi_{n}\frac{n_{\infty }\left(x\right)-n_{\infty }\left(y\right)}{\tau_{n}\left(x\right)n_{\infty }^{\prime }\left(y\right)}\\ \;+\rho_{p}\phi_{p}\frac{p_{\infty }\left(x\right)-p_{\infty }\left(y\right)}{\tau_{p}\left(x\right)p_{\infty }^{\prime }\left(y\right)}. \end{array}$$

In this study, we have used the approximations *v*_*h*_ ≈ *y*, *v*_*n*_ ≈ *y*, and *v*_*p*_ ≈ *y*.

For the variable *z*, based on Equation (11), its dynamics is written as,


(22) $$\begin{array}{l} \frac{dz}{dt}=\phi_{q}\frac{q_{\infty }\left(x\right)-q_{\infty }\left(z\right)}{\tau_{q}\left(x\right)q_{\infty }^{\prime }\left(z\right)}. \end{array}$$

The above Equations (20–22) form the reduced model of TRN neurons. The details of the model are given in Supplementary Material: The details of the reduced model.

## 4. Analysis of the Reduced Model

In the above, we have reduced the original six-variable TRN neuron model into a dynamical system with three variables. In this section, we investigate the firing patterns and perform a bifurcation analysis of the reduced model. All simulations and analyses are performed using the BrainPy library (Wang et al., 2021).

### 4.1. Firing Patterns in the Reduced Model

**Figure 2:**
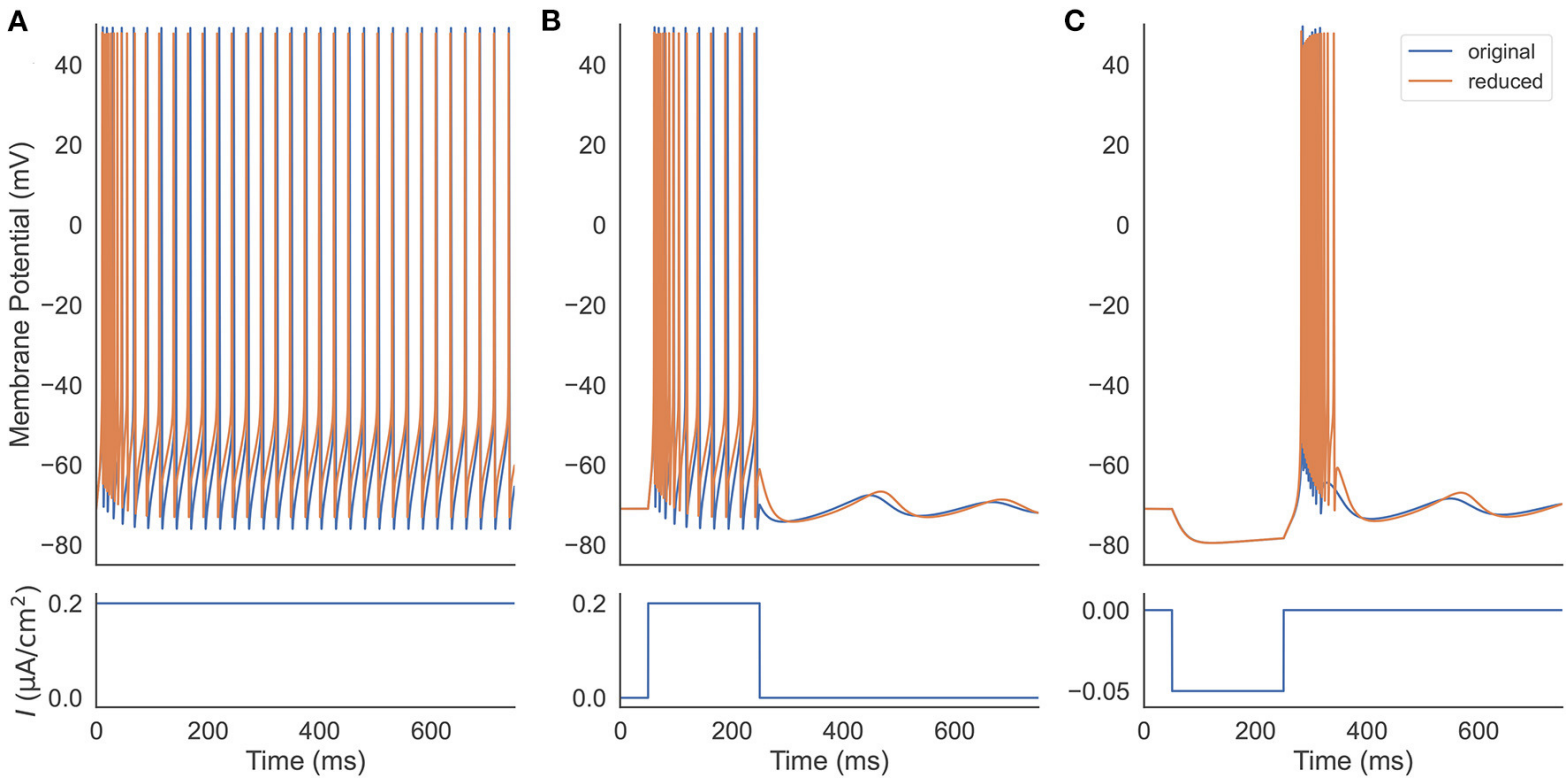
Firing pattern comparison between the original (Equation 1) and the reduced (Equations 20–22) TRN neuron model. **(A)** Two models are stimulated with a constant excitatory input 0.2 μA/cm^2^. They both exhibit decelerated burst and stationary tonic spikes. **(B)** Two models receive a step current with the size of 0.2 μA/cm^2^. The removal of the external current results in a similar subthreshold oscillation in both models. **(C)** An inhibitory current with the size of −0.05 μA/cm^2^ and the duration of 200 ms is applied to two models. Both rebound bursting and subthreshold oscillation observed in the full model are reproduced in the reduced one. The parameters used in this simulation are *g*_*T*_ = 2.25 mS/cm^2^, *g*_*KL*_ = 0.0152 mS/cm^2^, and *k* = 0.01.

We first verify that our reduced TRN neuron model behaves similarly to the original full one and can reproduce the firing patterns as seen in experiments. Two models are set to have the same parameters, including the neuronal parameters and the external input currents (Figure 2). Under a constant excitatory input with the size of 0.2 μA/cm^2^, two models first burst, then gradually produce the same tonic spikes (Figure 2A). After shortening the depolarization duration (the excitatory current is only applied during the first 200 ms, Figure 2B), the reduced model also shows the same oscillation behavior. Specifically, both models first burst, then display tonic spikes, and finally exhibit subthreshold oscillations. Moreover, injection of a hyperpolarizing current pulse (an inhibitory current with the size of −0.05 μA/cm^2^ and the duration of 200 ms) results in a similar rebound bursting (Figure 2C). Therefore, the reduced model can effectively capture the TRN neuron firing patterns, including tonic and burst firings, as seen in TRN neurons *in vivo* and *in vitro* (Contreras et al., 1992; Bal and McCormick, 1993; Lee et al., 2007).

In the next, we are going to apply dynamical system theory to illustrate the bifurcation mechanism underlying such firing patterns. We consider a fast-slow decomposition (Rinzel, 1985; Rinzel and Lee, 1987) based on the time scale differences between three variables. Specifically, in the reduced model (Equations 20–22), *V* and *y* are fast variables, and *z* is a slow variable.

### 4.2. Current-Voltage Relations in the Reduced Model

**Figure 3:**
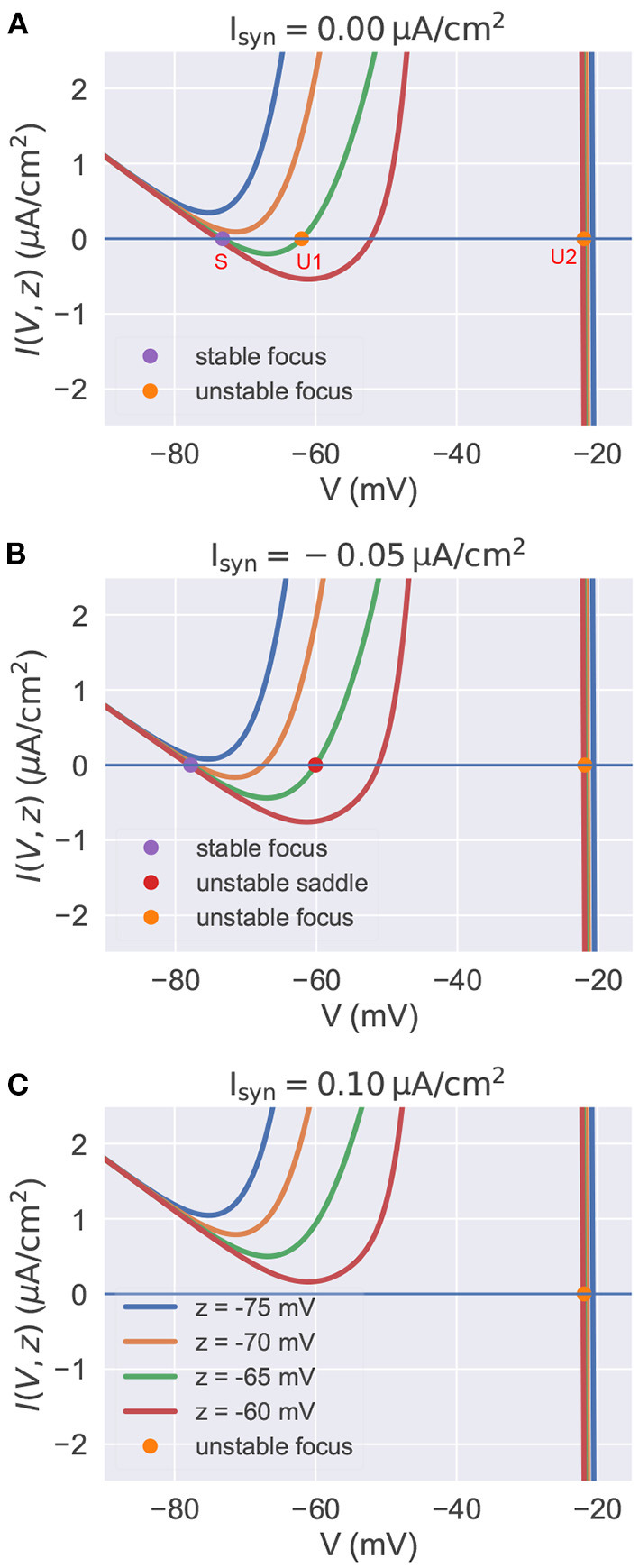
The current-voltage relation in the reduced model. In each panel, we plot multiple I-V curves with different values of *z*. I-V relations under the different settings of *I*_*syn*_ are plotted in **(A–C)** separately. The colored points indicate the fixed points when *z* is set to –65 mV. Other fixed points under the different settings of *z* are similar with these of *z* = −65 mV, so we omit them in the figure. **(A)** I-V curves when $I_{syn}=0.\mu \text{A}/\text{c}{\text{m}}^{2}$. For the high value of *z*, the model has three equilibrium points. **(B)** I-V curves when $I_{syn}=-0.05\;\mu \text{A}/\text{c}{\text{m}}^{2}$. The injection of inhibitory current results in the lower value of resting potential *V*. **(C)** I-V curves when $I_{syn}=0.10\;\mu \text{A}/\text{c}{\text{m}}^{2}$. Depolarizing currents shift the I-V curves upward, making the model only have one isolated unstable point associated with a stable limit cycle (corresponds to the action potential). The neuronal parameters used in this study are $g_{T}=2.25\text{ mS}/\text{c}{\text{m}}^{2}$, $g_{KL}=0.0065\text{ mS}/\text{c}{\text{m}}^{2}$, and *k* = 0.01.

In this section, by treating the slow variable *z* as a parameter, we analyze the stability of fixed points with the current-voltage (I-V) relation in the reduced model. In the parameter space of the biophysical condition, we obtain *y*(*t*) = *V*(*t*) by solving *dy*/*dt* = 0 in Equation (21). Substituting it into Equation (20), we get the I-V relation *I*(*V, z*) (the full equation, please refer to Supplementary Material: Current-voltage relations). In a voltage-clamp experiment, the *I*(*V, z*) measures under the given value of *z* the membrane current needs to inject to clamp the potential to the value of *V*. If *I*(*V, z*) = 0, the neuron is in the rest or equilibrium state at the value of *V*. The I-V curve of the reduced model is plotted in Figure 3 for various values of *z* and *I*_*syn*_. The equilibrium points are only plotted for *z* = −65 mV because other curves with different values of *z* have similar fixed points. The stability of each equilibrium point is evaluated by the linear stability analysis (refer to Supplementary Material: Linear stability analysis).

Figure 3A presents the I-V curves when no external input is applied. It reveals that for a high value of *z*, there are three equilibrium points: a stable focus (S) at the low membrane potential which corresponds to the resting potential, and two unstable fixed points (U1 and U2) with high potential values. Usually, the unstable focus (U2) with the highest potential value (near –20 mV) is surrounded by a stable limit cycle attractor. This means the system will exhibit bistability for a high value of *z*. Gradually decreasing *z*, the stable equilibrium point S and the unstable point U1 get close to each other, coalesce at a saddle-node bifurcation point, and then disappear. This makes the system display rapid action potentials because only the unstable point U2 and its associated limit cycle are left when the value of *z* is low.

Injection of the external current changes the position of I-V curves relative to the zero line *I* = 0. Figures 3B,C show the I-V curves of the reduced model when the inhibitory and excitatory currents are applied, respectively. Biophysically, the inhibitory current usually hyper-polarizes the membrane potential to a lower value. This can be interpreted by the I-V curves shown in Figure 3B. The inhibitory current moves the I-V curves downward, making the resting point S left-shifted. Moreover, the phenomenon that the injection of excitatory current depolarizes the neuron to produce action potentials can also be qualitatively understood by the I-V curve shown in Figure 3C. Because depolarizing currents shift the curves upward, eliminating the bistable property of the model and leaving an isolated unstable point surrounded by a stable limit cycle (corresponds to the action potential).

### 4.3. Rebound Bursting *via* the “Fold/Homo-Clinic” Bifurcation

The I-V curves above let us inspect the neuronal excitability under the different values of *z* and *I*_*syn*_. However, the mechanism of why the model produces rebound bursting and tonic spiking is still not clear. By continually treating the slow variable *z* as a bifurcation parameter, in the next two sections, we analyze the phase portraits of the fast subsystem, and then interpret the model as the evolution of the phase portraits under the control of the slow variable *z*.

**Figure 4:**
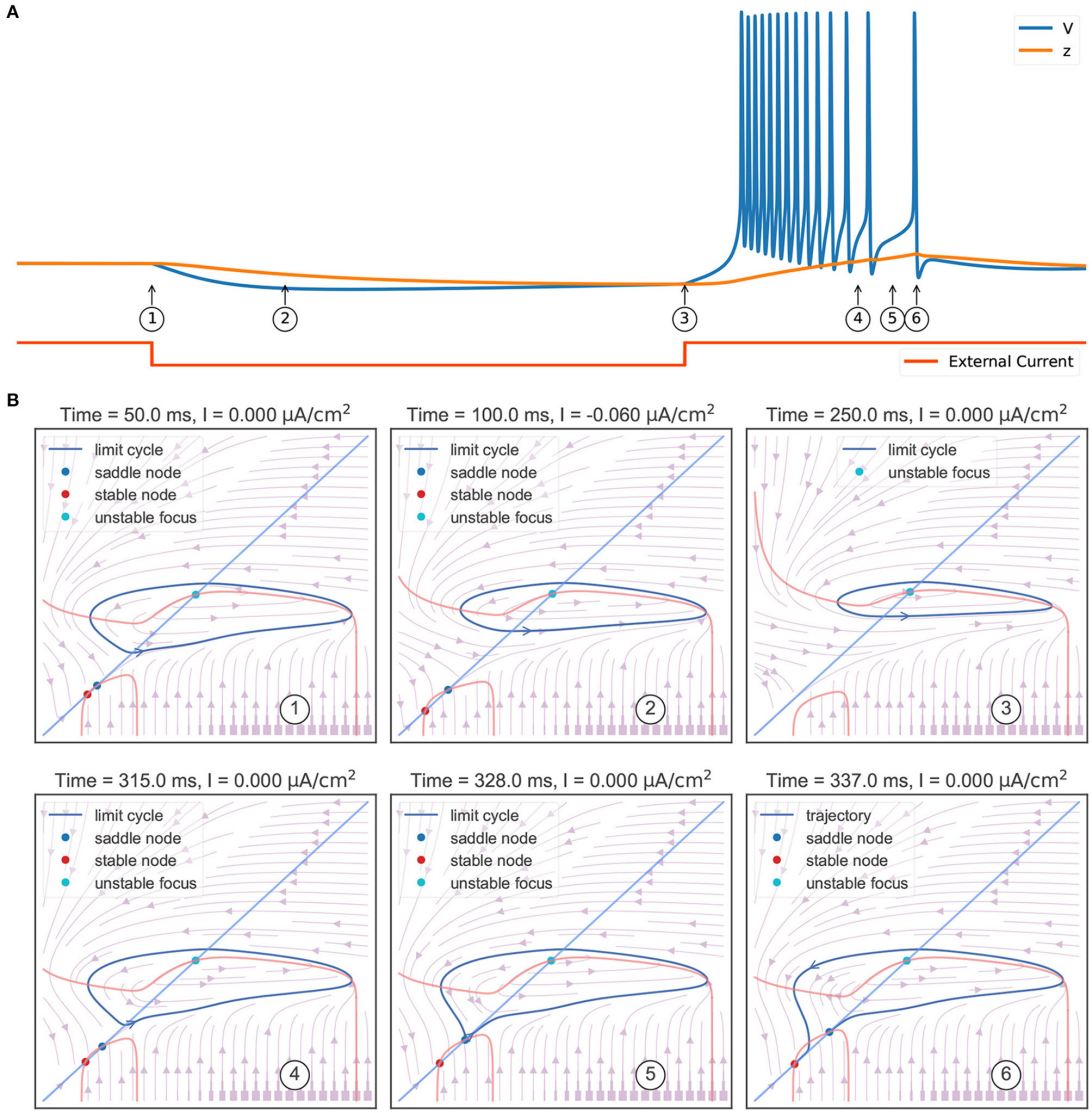
Phase plane analysis during the TRN rebound bursting. **(A)** The bursting behavior of the three-variable TRN model after receiving an inhibitory current. The external input with the size of −0.03 mS/cm^2^ is given during 50–250 ms. **(B)** Transitions of phase portraits as the slow variable changes over time. ①: Without the external input, the model is bistable. Under the initial condition, the model is in the resting state (corresponds to the stable node). ②: Applying an inhibitory current makes us get smaller values of resting potential *V* and slow variable *z*. ③: Once upon the current is removed, only the unstable focus and its associated limit cycle (action potentials) are left. ④: Repetitive action potentials increase the slow variable *z* and make the model bistable again. ⑤: Once *z* is so large that the limit cycle becomes a homo-clinic orbit to a saddle and the repetitive spiking cannot be sustained. ⑥: When *z* reaches its highest value, the model directly jumps to its global stable node. The neuronal parameters used in this study are $g_{T}=2.25\text{ mS}/\text{c}{\text{m}}^{2}$, $g_{KL}=0.0065\text{ mS}/\text{c}{\text{m}}^{2}$, and *k* = 0.01.

The behavior sequence in a rebound bursting (Figure 4A) when the TRN neuron receives a hyperpolarizing current is illustrated in Figure 4B. At the resting potential without external input (①), the model exhibits a bistable behavior: a stable node (corresponding to the resting potential), a saddle-node, and an unstable node with a limit cycle. Once a hyperpolarizing current *I* = −0.06 μA/cm^2^ is applied (②), the value of the stable node starts to decline, resulting in lower values of the membrane potential *V* and the slow variable *z*. Further, the withdrawal of such inhibitory current (③) removes the stable equilibrium state, with only an unstable focus left (associated with a limit cycle). This leads the model to produce rapid action potential and gradually increase the slow variable value *z*. The increasing *z* once again makes the model bistable (④). However, the model is still in the stable limit cycle state due to the hysteresis. The repetitive spiking stops when *z* is too large. This happens when *z* passes the value of –63.25 mV (⑤), the limit cycle becomes a homo-clinic orbit to a saddle, and then disappears. When *z* increases to the highest value (⑥), the bursting trajectory jumps down to the stable equilibrium corresponding to the resting state (stable node). Therefore, according to the bursting classification schema of Izhikevich (2007), the firing pattern exhibited in the TRN neuron model should be named the “fold/homo-clinic” bursting, because the resting state disappears through the fold bifurcation (② → ③), then the spiking limit cycle state disappears through the saddle homo-clinic orbit bifurcation (⑤).

**Figure 5:**
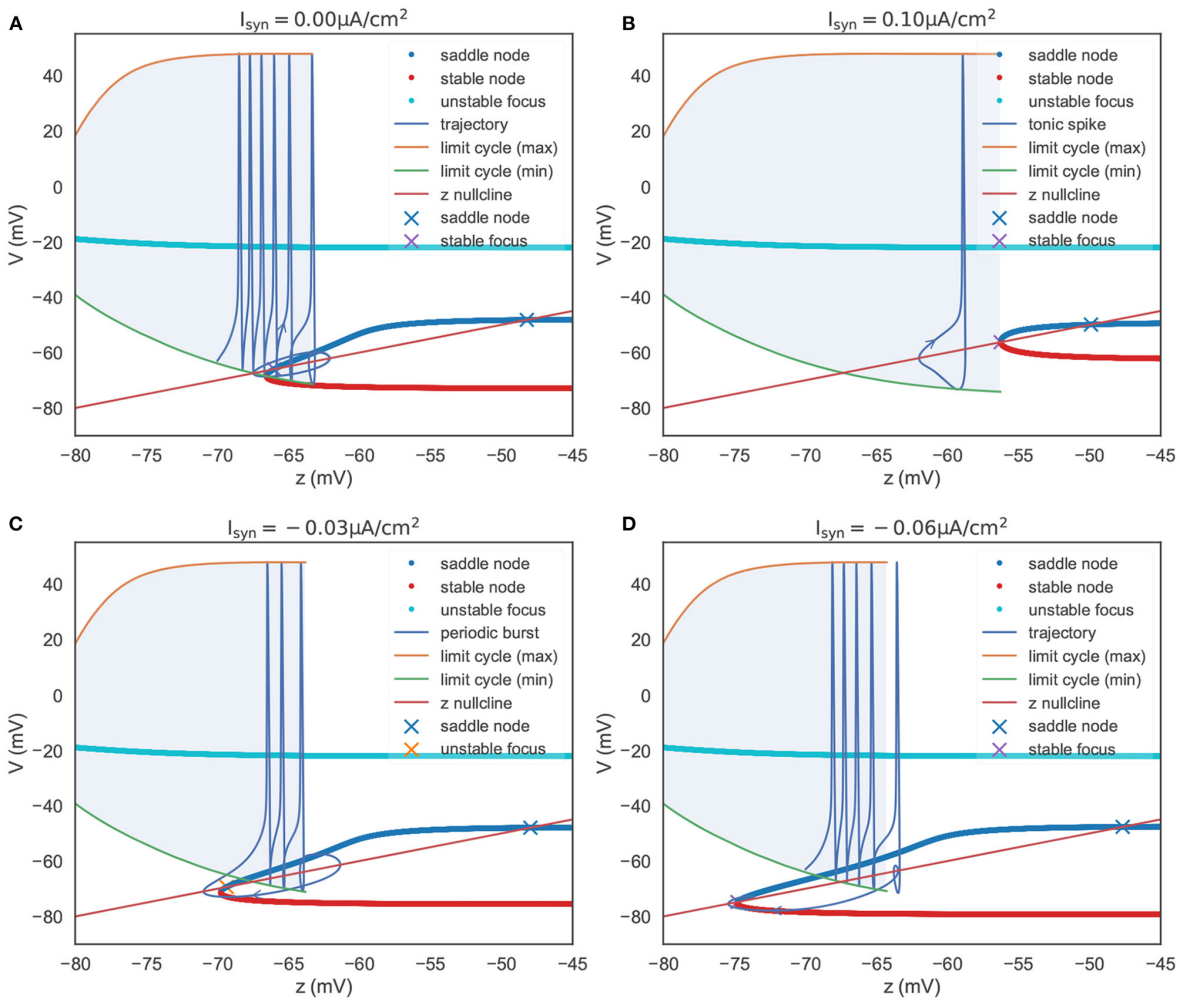
The bifurcation diagram of the TRN neuron. The colored dots indicate the fixed points evaluated in the *V*-*y* two-dimensional sub-system (refer to Figure 4B). They constitute the slow manifold. The “x” dots, intersections of the slow manifold and the *z* nullcline, denote the global fixed points evaluated in the *V*-*y*-*z* system. **(A)** The bifurcation structure when $I_{syn}=0.0\;\mu \text{A}/\text{c}{\text{m}}^{2}$. When *z* is small, the model produces a barrage of action potentials, i.e., bursting. Meanwhile, *z* increases until it reache *z* = –63.33 mV, where a saddle homo-clinic orbit bifurcation occurs. Then *z* decreases due to *dz*/*dt* < 0, and the trajectory terminates due to the attraction of the global stable focus. **(B)** The bifurcation structure when $I_{syn}=0.1\;\mu \text{A}/\text{c}{\text{m}}^{2}$. The depolarizing current changes the bifurcation structure, and results in two distinct manifolds. The first is a region where an unstable focus is surrounded by a stable limit cycle. The second is a regime in which multiple fixed points coexist. During the first regime, the decrease of *z* (*dz*/*dt* < 0) exactly balances the increase of *z* (*dz*/*dt* > 0), leading the model to have the ability to produce tonic spiking. **(C)** The bifurcation structure when $I_{syn}=-0.03\;\mu \text{A}/\text{c}{\text{m}}^{2}$. Under the weak input, the model to have the ability to produce periodic bursts. **(D)** The bifurcation structure when $I_{syn}=-0.06\;\mu \text{A}/\text{c}{\text{m}}^{2}$. When the hyper-polarization current is strong, the model will jump to the stable focus state after a homo-clinic bifurcation. The parameters used in this figure are $g_{KL}=0.0065\text{ mS}/\text{c}{\text{m}}^{2}$, $g_{T}=2.25\text{ mS}/\text{c}{\text{m}}^{2}$, and *k* = 0.01.

To summarize the phase portraits shown in Figure 4B, a bifurcation analysis for the rebound bursting concerning the slow variable *z* is presented in Figure 5A. For *z* < −66.54 mV, the model only exhibits stable action potentials. The amplitude of action potential decreases with the smaller value of *z*. However, for −66.54 mV ≤ *z* ≤ −63.33 mV, the model exhibits bi-stability due to the emergence of a stable node from the fold bifurcation. The saddle-node denotes the separation of attraction regions between two stable attractors. For *z* > −63.33 mV, only the stable nodes are exhibited due to the disappearance of the action potential through the saddle homo-clinic orbit bifurcation.

In Figure 5A, we also plot the *z* nullcline *V*(*t*) = *z*(*t*), which is obtained by solving *dz*/*dt* = 0 in Equation (22). The intersections of the *z* nullcline and the bifurcation structure, denoted by the “ × ” symbols, are evaluated by the linear stability analysis (refer to Supplementary Material: Linear stability analysis). When initialized in the attraction domain of limit cycles, the model produces a barrage of action potentials (refer to the “trajectory” line in Figure 5A). During the spike train of action potentials, *z* increases until it reaches a point (*z* = −63.33 mV) that corresponds to the saddle homo-clinic orbit bifurcation. Then, the trajectory is attracted toward the stable nodes (red dots in Figure 5A), and *z* decreases due to *dz*/*dt* < 0. *z* decreases until it goes across the *z* nullcline, and *V* starts to increase when a fold bifurcation occurs at *z* = −66.54 mV. Finally, the trajectory will terminate at the lowest intersection, which is a stable focus point.

### 4.4. Tonic Spiking Due to the Fold Cycle Bifurcation

**Figure 6:**
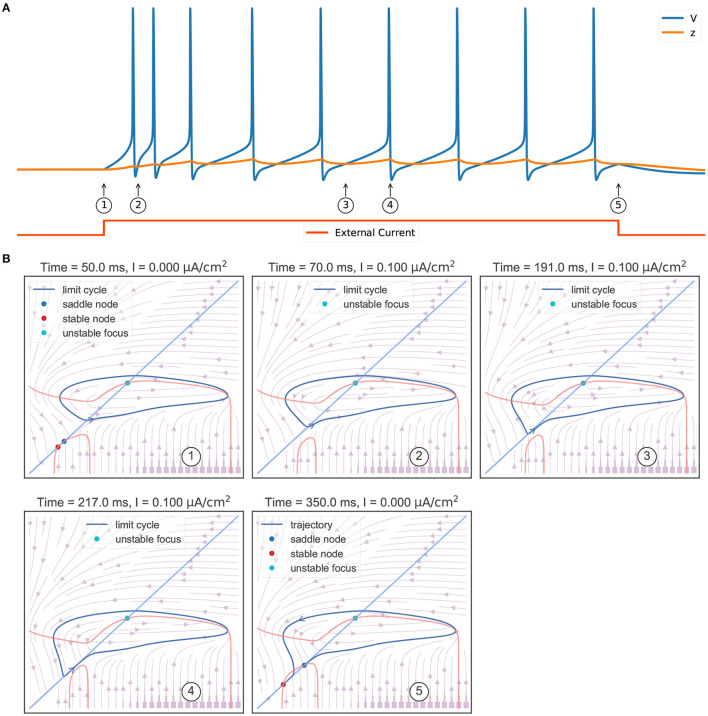
Phase plane analysis of the TRN tonic spiking. **(A)** The model behavior when receiving a constant excitatory input. The external current with the size of 0.1 mS/cm^2^ is injected during 50–350 ms. The model first shows transient decelerated burst and then displays tonic spikes. **(B)** The evolution of phase portraits as the slow variable changes in time. ①: Without the external input, the model exhibits bistable behavior. Under the initial condition, the model is in the resting state and has a small value of slow variable *z*. ②: The excitatory input switches the bistable state to an unstable focus associated with a stable limit cycle. Due to the initial slow variable, *z* is small, the model first exhibits a barrage of fast action potentials. ③ and ④: Once variable *z* reaches to big value, the potential *V* and the slow variable *z* co-evolve with each other, and the model exhibits stable action potentials. ⑤ When the input is withdrawn, a global stable state emerges and the model trajectory jumps to the resting state. The parameters used in this study are $g_{T}=2.25\text{ mS}/\text{c}{\text{m}}^{2}$, $g_{KL}=0.0065\text{ mS}/\text{c}{\text{m}}^{2}$, and *k* = 0.01.

Another key feature of the TRN neuron is the tonic spiking (Figure 6A) during a long-lasting excitation. Figure 6B shows the corresponding phase portraits. Same as Figure 4B, in the resting state (without external input) the model has a bistable behavior, in which a stable node and a stable limit cycle coexist (①). Then, the injection of an excitatory current (②) destroys this bi-stability property, making the model only have a stable action potential attractor. Since the slow variable *z*, which is initially small, gradually increases, the model exhibits the decelerated burst discharges at the beginning of the current injection (②). Once *z* is big enough, the slow variable *z* will show periodic orbit with the same frequency as the fast variable *V* (③ and ④). This stable periodic solution, or the tonic spiking, will last until the external excitatory current is removed (⑤). Because the removal of the excitatory current will change the distribution of fixed points in the model, leaving a global stable node corresponding to the resting state (⑤).

Figure 5B summarizes the phase portraits during a TRN tonic spiking. Different from the bifurcation diagram of a rebound bursting (Figure 5A), when an excitatory current with the size of 0.1 μA/cm^2^ is injected, the model displays two distinct manifolds. The first is a region in which the model has an unstable focus surrounded by a stable action potential limit cycle when *z* is small. This manifold region terminates after a fold limit cycle bifurcation occurs at *z* = −56.26 mV. Then the second region appears in which multiple fixed points coexist. In this manifold, the stable limit cycle disappears, only leaving a stable node corresponding to the resting potential.

### 4.5. One-Parameter Bifurcation Analysis

In the above, we have investigated the bifurcation mechanism of the rebound bursting and the tonic spiking by a zero current and an excitatory current, respectively. In the next, we continue to inspect the neuronal excitability by varying the size of external currents.

Figure 5C presents the bifurcation diagram of the TRN neuron model when given an inhibitory current with the size of −0.03 μA/cm^2^. As before, *z* will increase until *z* = −63.77 mV, whereupon the action potentials disappear through a saddle homo-clinic orbit bifurcation. *z* will continue to increase until the trajectory passes through the *z* nullcline. Then the system moves toward the stable node solution, and *z* decreases due to *dz*/*dt* < 0 until the stable node disappears when the saddle-node bifurcation occurs at *z* = −69.775 mV. Different from the bifurcation diagram shown in Figure 5A, the lowest intersection of the *z* nullcline and the slow manifold is an unstable focus, making the system moves away to the attraction domain of limit cycles again. The critical feature in Figure 5C is the bi-stability between two stable solutions (the limit cycle and the stable node) for *z* between two bifurcations (the saddle-node bifurcation and the saddle homo-clinic orbit bifurcation). It is this bi-stability that is important for the periodic bursting behavior shown in Figure 5C. Moreover, the length of the bistable interval determines the number of spikes in a burst.

However, with the increasing value of the inhibitory current, the unstable intersection becomes stable. In Figure 5D, we present the bifurcation diagram when the current of −0.06 μA/cm^2^ is injected. This time *z* increases until *z* = −64.245 mV, and the fold bifurcation occurs at *z* = −74.8 mV. Once the limit cycle disappears through the homo-clinic bifurcation, the trajectory will directly jump to the steady-state where the *z* nullcline intersects the slow manifold at −74.776 mV.

**Figure 7:**
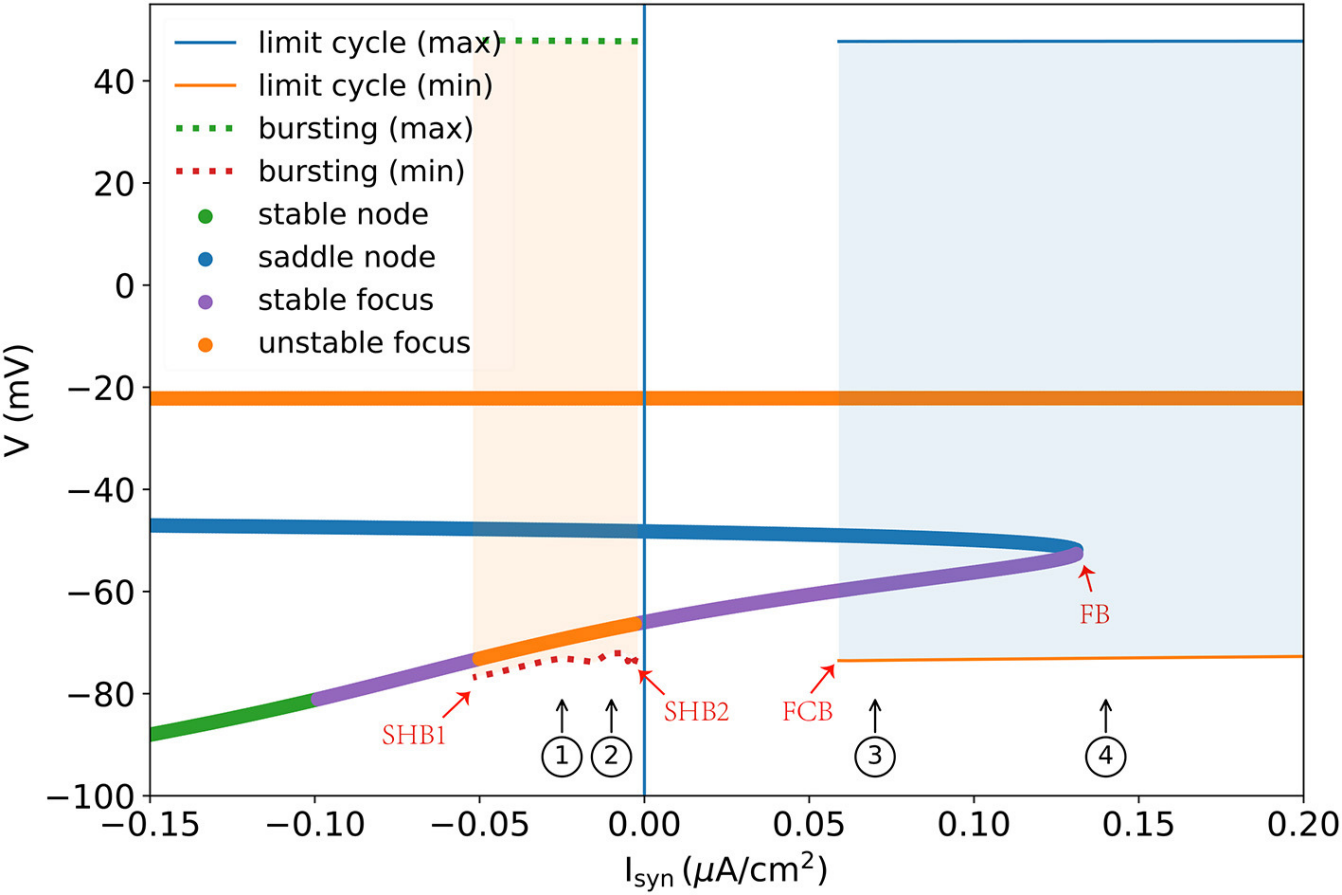
One parameter bifurcation diagram of the TRN neuron model. “SHB” denotes the super-critical Andronov-Hopf bifurcation, “FCB” presents the fold cycle bifurcation, and “FB” indicates the fold bifurcation. The dotted line is the maximum and minimum values of *V* during the periodic burst, and the solid line (—) indicates the maximum and minimum values of *V* during the limit cycle. Every colored dot is evaluated by the linear stability analysis of the *V*-*y*-*z* system. For the detalied explanation please refer to the main text. The parameters used are $g_{KL}=0.0065\text{ mS}/\text{c}{\text{m}}^{2}$, $g_{T}=2.25\text{ mS}/\text{c}{\text{m}}^{2}$, and *k* = 0.01.

**Figure 8:**
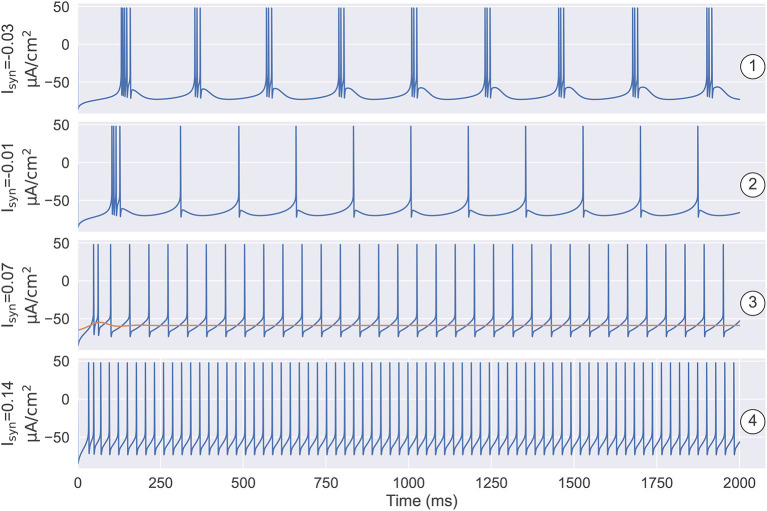
The simulation results under the different settings of *I*_*syn*_ and the initial state. ① and ② illustrate that the number of spikes in a burst is determined by the size of the inhibitory current. ①: when $I_{syn}=-0.03\;\mu \text{A}/\text{c}{\text{m}}^{2}$, the model produces the robust periodic bursting in which each burst has three spikes. ②: decreasing the inhibitory current to $I_{syn}=-0.01\;\mu \text{A}/\text{c}{\text{m}}^{2}$, the number of spikes in a burst decreases to one. ③ demonstrates the model has the bistable property when depolarizing current *I*_*syn*_ is small. One is the stable resting state, the other is the stable limit cycle. Specifically, when $I_{syn}=0.07\;\mu \text{A}/\text{c}{\text{m}}^{2}$, models with different initial states are evolved to different attractors. ④ illustrates that once the external input is big enough, i.e., $I_{syn}=0.14\;\mu \text{A}/\text{c}{\text{m}}^{2}$, the model only has a stable limit cycle attractor, which corresponds to the tonic spiking mode as seen in the TRN neuron. The parameters used in this figure are $g_{KL}=0.0065\text{ mS}/\text{c}{\text{m}}^{2}$, $g_{T}=2.25\text{ mS}/\text{c}{\text{m}}^{2}$, and *k* = 0.01.

A one-parameter bifurcation diagram summarizing the change in the model dynamics with the external input I_syn_ is shown in Figure 7. For ${\text{I}}_{\text{syn}}<-0.052\;\mu \text{A}/\text{c}{\text{m}}^{2}$, the system has a steady-state as previously demonstrated in Figure 5D. As I_syn_ increases, a super-critical Andronov-Hopf bifurcation (SHB) occurs at ${\text{I}}_{\text{syn}}=-0.052\;\mu \text{A}/\text{c}{\text{m}}^{2}$ (point “SHB1” in Figure 7) due to a stable equilibrium state changes to a stable periodic orbit along with a two-dimensional unstable manifold. The stable orbit exhibited in the model is the periodic bursting (the red and green dotted lines ⋯ in Figure 7). This corresponds to the situation in Figure 5C, where the bi-stability between two bifurcations leads the system to have a periodic bursting solution. The number of the spike in a burst is determined by the size of the inhibitory current. Specifically, the smaller size of the injected inhibitory input, fewer spikes in a burst (refer to ① and ② in Figures 7, 8). Intuitively understanding, this is because the higher value of inhibitory current can drive the neuron into a more hyperpolarized state. The more the neuron is hyperpolarized, the low-threshold calcium channel *I*_*T*_ is more deinactivated, further resulting in the neuron producing stronger bursting. Further increasing I_syn_ makes the model stable again *via* another SHB at ${\text{I}}_{\text{syn}}=-0.003\;\mu \text{A}/\text{c}{\text{m}}^{2}$ (point “SHB2” in Figure 7). The post-inhibitory excitation current with the size in this parameter region (specifically, $-0.003\;\mu \text{A}/\text{c}{\text{m}}^{2}<{\text{I}}_{\text{syn}}<0.059\;\mu \text{A}/\text{c}{\text{m}}^{2}$) will result in a rebound bursting as illustrated in Figures 4, 5A. 0.059 μA/cm^2^ is the minimum excitation current necessary to trigger an action potential of the model, because the stable limit cycle occurs after a fold cycle bifurcation appears at ${\text{I}}_{\text{syn}}=0.059\;\mu \text{A}/\text{c}{\text{m}}^{2}$ (point “FCB” in Figure 7). This corresponds to the situation shown in Figures 5B, 6 where the increase of *z* during the trajectory is above the *z* nullcline exactly balances the decrease of *z* during the trajectory is below the *z* nullcline, making the sustained action potential occurs (the solid lines—in Figure 7). It is worth noting that during this parameter region (specifically $0.059\;\mu \text{A}/\text{c}{\text{m}}^{2}<{\text{I}}_{\text{syn}}<0.1316\;\mu \text{A}/\text{c}{\text{m}}^{2}$) the system is bistable, as a stable limit cycle and a stable-steady state coexist. This means the final state of the system depends on the initial conditions (refer to ③ in Figures 7, 8). The bistable mode continues until ${\text{I}}_{\text{syn}}=0.1316\;\mu \text{A}/\text{c}{\text{m}}^{2}$, where a fold bifurcation occurs (“FC” point in Figure 7). While the model still exhibits sustained action potentials (refer to example of ④ shown in Figures 7, 8).

## 5. Conclusion and Discussion

The thalamic reticular nucleus is a strategical locus due to its pacemaking role in spindle generation during sleep (Steriade et al., 1985, 1987) and its gatekeeper function in selective attention during wakefulness (Halassa et al., 2014; Wimmer et al., 2015; Nakajima et al., 2019). Understanding its intrinsic dynamics is an important step toward understanding its functions. Previously, TRN neurons were modeled using complex realistic conductance-based models whose dynamics are hard to analyze and visualize. In this study, we developed a reduced three-variable neuron model to capture the key dynamical features of TRN neurons. We demonstrated that the reduced model can effectively reproduce the characteristic firing patterns of TRN neurons (Figure 2), but its dynamics are much easier to be analyzed. The bifurcation diagrams illustrate the underlying mechanisms of the two characteristic TRN firing patterns, that is, the rebound bursting is “fold/homo-clinic” bifurcation (Figures 4, 5) and the tonic spiking is the fold cycle bifurcation (Figures 5, 6). Furthermore, one-parameter bifurcation analysis demonstrates that the model displays varying degrees of bursting and tonic discharges when the size of the external current changes (Figures 7, 8). We expect that this study will facilitate our future work to study the complicated dynamics of the TRN network.

It has been proved feasible that neuron models with high-dimensional voltage-gating variables by using the Hodgkin-Huxley schema can be described by a reduced system with fewer essential variables (Chay, 1985; Av-Ron et al., 1993; Golomb et al., 1993; Chay et al., 1995; Maeda et al., 1998; Izhikevich, 2007). The pioneering work is the FitzHugh-Nagumo model (FitzHugh, 1961; Nagumo et al., 1962; Izhikevich and FitzHugh, 2006). It provides a simplified model for the Hodgkin-Huxley model (Hodgkin and Huxley, 1952) and allows us to inspect the mechanism of neuronal excitability and spike generation from the geometrical viewpoints. Later, by the massive numerical simulation, Krinskii and Kokoz (1973) provided an empirical conclusion that gating variables *n* and *h* in the Hodgkin-Huxley model have a linear relationship: *n*(*t*) + *h*(*t*) ≈ 0.84. Thus, *h* and *n* variables can be approximated by a single variable. Inspired by this reduction idea, Abbott and Kepler (1990) and Kepler et al. (1992) suggested a more general method to reduce the Hodgkin-Huxley-type neuron models. The core concept behind this method is that: if gating variables behave in a similar time scale, a single variable can be obtained by the weighted average of them. Specifically, the weights should be the relative contributions to the overall membrane potential change. In this study, we applied this method to reduce a full TRN neuron model (Bazhenov et al., 1998, 2002) into a three-variable model. Discharge patterns are essential factors in neural information processing. Our reduction model can reproduce the firing patterns seen in the original model. This demonstrated our reduction is valid (Golomb et al., 1993; Maeda et al., 1998).

Since the seminal paper of Rinzel and Ermentrout (1998) introduced a geometrical method for phase plane analysis on neuronal models, phase plane analysis has become standard on neural modeling. Afterward, under the guidance of such dynamical system theory, simplified but powerful neuron models were proposed, such as the Izhikevich neuron model (Izhikevich, 2003, 2007) and adaptive exponential integrate-and-fire model (Brette and Gerstner, 2005; Gerstner and Brette, 2009). These low-dimensional abstract neuron models are capable of producing tonic spiking and bursting patterns. However, the neuronal parameters largely differ between firing patterns, making the continuous switch between different firing patterns (as seen in biological experiments Bal and McCormick, 1993; Llinás and Steriade, 2006; Halassa and Acsády, 2016) difficult. On the contrary, our proposed three-variable model which is reduced directly from the realistic conductance neuron model can effectively capture different firing patterns and the coherent switch between them (Figures 7, 8). The reduced three-variable model retains the fundamental biophysical properties of the original, as each channel dynamics can be easily recovered from the reduced variables [refer to Eq. (S22) in Supplemental Material].

## Data Availability Statement

The original contributions presented in the study are included in the article/Supplementary Material. All datasets generated for this study are included in the article. The source code for the simulation and analysis is available at https://github.com/chaoming0625/TRNNeuronAnalysis.

## Author Contributions

CW and SW designed the project. CW, SL, and SW performed the formal analysis and wrote the manuscript. CW carried out simulations. SW supervised the project. All authors contributed to the article and approved the submitted version.

## Funding

This study was supported by Guangdong Province with grant (no. 2018B030338001, SW) and Huawei Technology Co., Ltd. (no. YBN2019105137, SW).

## Conflict of Interest

The authors declare that the research was conducted in the absence of any commercial or financial relationships that could be construed as a potential conflict of interest.

## Publisher's Note

All claims expressed in this article are solely those of the authors and do not necessarily represent those of their affiliated organizations, or those of the publisher, the editors and the reviewers. Any product that may be evaluated in this article, or claim that may be made by its manufacturer, is not guaranteed or endorsed by the publisher.
