# Supplementary material for: Analysis of the Neuron Dynamics in Thalamic Reticular Nucleus by a Reduced Model
Source: Front Comput Neurosci. 2021 Nov 16;15:764153. doi: 10.3389/fncom.2021.764153 (PMC8635031; doi:10.3389/fncom.2021.764153)
Supplement: Supplementary file 1 [file Data_Sheet_1.pdf]

Supplementary Material

1 FIXING THE NEW VARIABLES

As mentioned in the main text, the determination of the coefficients for new variables in Eqs. (9-11) is formulated as an optimization problem. Specifically, for determining the coefficients $\rho_h, \rho_n, \rho_p, \rho_m, \rho_V$, the optimization problem is formulated as,

$$\min \quad \left| L(\delta_{v_h}, \delta_{v_n}, \delta_{v_p}, \delta_{v_m}, \delta_V) \right|, \tag{S1}$$

subject to the constraints,

$$\rho_h \delta_{v_h} + \rho_n \delta_{v_n} + \rho_p \delta_{v_p} = 0, \quad \rho_h, \rho_n, \rho_p \geq 0, \tag{S2}$$

$$\rho_V \delta_V + \rho_m \delta_{v_m} = 0, \quad \rho_m, \rho_V \geq 0, \tag{S3}$$

where

$$\begin{aligned} L(\delta_{v_h}, \delta_{v_n}, \delta_{v_p}, \delta_{v_m}, \delta_V) =& \delta_{v_h} \rho_h \frac{\partial F}{\partial v_h} + \delta_{v_n} \rho_n \frac{\partial F}{\partial v_n} + \delta_{v_p} \rho_{v_p} \frac{\partial F}{\partial v_p} + \\ & \delta_V \left(\rho_V \frac{\partial F}{\partial V} - \frac{C\phi_m \rho_m}{\tau_m} \right) + \delta_{v_m} \left(\rho_V \frac{\partial F}{\partial v_m} + \frac{C\phi_m \rho_m}{\tau_m} \right). \end{aligned} \tag{S4}$$

To simplify the calculation, we ignore the constraints $\rho_h, \rho_n, \rho_p \geq 0$ and $\rho_m, \rho_V \geq 0$ in the optimization. However, since v_h and v_n are anti-synergistic to v_p, in term of that the increases of v_h and v_n hyper-polarize the membrane potential while the increase of v_p depolarizes the potential, it was pointed out in Kepler et al. (1992) that the optimization can lead to negative values of the coefficients. To avoid this, we fix ρ_p to a positive constant, i.e., $\rho_p = k$, with $0 \leq k \leq 1$, and only optimize ρ_h and ρ_n. This kind of method has been successfully applied to reduce the complex *Onchidium* pacemaker neuron model to a three-variable system (Maeda et al., 1998). Then, the optimization problem is re-written as,

$$\begin{aligned} \min \quad & \left| L(\delta_{v_h}, \delta_{v_n}, \delta_{v_p}, \delta_{v_m}, \delta_V) \right|, \\ \text{s.t.} \quad & \rho_h \delta_{v_h} + \rho_n \delta_{v_n} + k \delta_{v_p} = 0, \\ & \rho_V \delta_V + \rho_m \delta_{v_m} = 0. \end{aligned} \tag{S5}$$

Since the term to be minimized takes its absolute value, we differentiate two situations. The first situation is when $L(\delta_{v_h}, \delta_{v_n}, \delta_{v_p}, \delta_{v_m}, \delta_V) \geq 0$, and the optimization problem becomes,

$$\begin{aligned} \min \quad & L(\delta_{v_h}, \delta_{v_n}, \delta_{v_p}, \delta_{v_m}, \delta_V) \\ \text{s.t.} \quad & \rho_h \delta_{v_h} + \rho_n \delta_{v_n} + k \delta_{v_p} = 0, \\ & \rho_V \delta_V + \rho_m \delta_{v_m} = 0. \end{aligned} \tag{S6}$$

We apply the Lagrangian method to solve the optimization problem, and the corresponding Lagrangian function is constructed as,

$$\begin{aligned} H_1(\delta_{v_h}, \delta_{v_n}, \delta_{v_p}, \delta_{v_m}, \delta_V, \lambda_1, \lambda_2) =& L(\delta_{v_h}, \delta_{v_n}, \delta_{v_p}, \delta_{v_m}, \delta_V) + \\ & \lambda_1(\rho_h \delta_{v_h} + \rho_n \delta_{v_n} + k\delta_{v_p}) + \lambda_2(\rho_m \delta_{v_m} + \rho_V \delta_V). \end{aligned} \tag{S7}$$

Taking the partial derivatives of $H_1(\delta_{v_h}, \delta_{v_n}, \delta_{v_p}, \delta_{v_m}, \delta_V, \lambda_1, \lambda_2)$ to variables $\delta_{v_h}, \delta_{v_n}$ to be zeros, we have,

$$\frac{\partial H_1}{\partial \delta_{v_h}} = \rho_V \frac{\partial F}{\partial v_h} + \lambda_1 \rho_h = 0, \tag{S8}$$

$$\frac{\partial H_1}{\partial \delta_{v_n}} = \rho_V \frac{\partial F}{\partial v_n} + \lambda_1 \rho_n = 0. \tag{S9}$$

Further using the condition $\rho_h + \rho_n + k = 1$, we obtain,

$$\rho_h =(1-k)\frac{\partial F}{\partial v_h} / \left(\frac{\partial F}{\partial v_h} + \frac{\partial F}{\partial v_n} \right), \tag{S10a}$$

$$\rho_n =(1-k)\frac{\partial F}{\partial v_n} / \left(\frac{\partial F}{\partial v_h} + \frac{\partial F}{\partial v_n} \right). \tag{S10b}$$

The other situation is when $L(\delta_{v_h}, \delta_{v_n}, \delta_{v_p}, \delta_{v_m}, \delta_V) < 0$, and the optimization problem can be written as,

$$\begin{aligned} \min \quad & -L(\delta_{v_h}, \delta_{v_n}, \delta_{v_p}, \delta_{v_m}, \delta_V), \\ \text{s.t.} \quad & \rho_h \delta_{v_h} + \rho_n \delta_{v_n} + k\delta_{v_p} = 0, \\ & \rho_V \delta_V + \rho_m \delta_{v_m} = 0. \end{aligned} \tag{S11}$$

The corresponding Lagrangian function is given by

$$\begin{aligned} H_2(\delta_{v_h}, \delta_{v_n}, \delta_{v_p}, \delta_{v_m}, \delta_V, \lambda_3, \lambda_4) =& - L(\delta_{v_h}, \delta_{v_n}, \delta_{v_p}, \delta_{v_m}, \delta_V) + \\ & \lambda_3(\rho_h \delta_{v_h} + \rho_n \delta_{v_n} + k\delta_{v_p}) + \lambda_4(\rho_m \delta_{v_m} + \rho_V \delta_V). \end{aligned} \tag{S12}$$

Taking the partial derivatives of $H_2(\delta_{v_h}, \delta_{v_n}, \delta_{v_p}, \delta_{v_m}, \delta_V, \lambda_3, \lambda_4)$ to the corresponding variables $\delta_{v_h}, \delta_{v_n}$ to be zeros, we have,

$$\frac{\partial H_2}{\partial \delta_{v_h}} = -\rho_V \frac{\partial F}{\partial v_h} + \lambda_3 \rho_h = 0, \tag{S13}$$

$$\frac{\partial H_2}{\partial \delta_{v_n}} = -\rho_V \frac{\partial F}{\partial v_n} + \lambda_3 \rho_n = 0. \tag{S14}$$

Using the condition $\rho_h + \rho_n + k = 1$, we obtain,

$$\rho_h =(1-k)\frac{\partial F}{\partial v_h} / \left(\frac{\partial F}{\partial v_h} + \frac{\partial F}{\partial v_n} \right), \tag{S15a}$$

$$\rho_n =(1-k)\frac{\partial F}{\partial v_n} / \left(\frac{\partial F}{\partial v_h} + \frac{\partial F}{\partial v_n} \right). \tag{S15b}$$

We see that in both situations, we reach the same solution. Furthermore, since $\frac{\partial F}{\partial v_h}$ and $\frac{\partial F}{\partial v_n}$ have the same sign, ensuring that the constrain $\rho_h, \rho_n, \geq 0$ is satisfied. In summary, the values of ρ_p, ρ_h, ρ_n are given by,

$$\rho_p = k, \tag{S16a}$$

$$\rho_h = (1-k)\frac{\partial F}{\partial v_h} / \left(\frac{\partial F}{\partial v_h} + \frac{\partial F}{\partial v_n}\right), \tag{S16b}$$

$$\rho_n = (1-k)\frac{\partial F}{\partial v_n} / \left(\frac{\partial F}{\partial v_h} + \frac{\partial F}{\partial v_n}\right), \tag{S16c}$$

In practice, we empirically choose k to best match the trajectory of the reduced model with that of the original full model under various sizes of the external current.

For determining the coefficients ρ_V, ρ_m, by taking the partial derivative of $H_1(\delta_{v_h}, \delta_{v_n}, \delta_{v_p}, \delta_{v_m}, \delta_V, \lambda_1, \lambda_2)$ to variables δ_V, δ_{v_m} to zeros, we have,

$$\frac{\partial H_1}{\partial \delta_V} = \rho_V \frac{\partial F}{\partial V} - \frac{C\phi_m \rho_m}{\tau_m} + \lambda_2 \rho_V = 0, \tag{S17a}$$

$$\frac{\partial H_1}{\partial \delta_{v_m}} = \rho_V \frac{\partial F}{\partial v_m} + \frac{C\phi_m \rho_m}{\tau_m} + \lambda_2 \rho_m = 0. \tag{S17b}$$

From the above equations, we get,

$$\lambda_2 = -\rho_V \frac{\partial F}{\partial V} - \rho_V \frac{\partial F}{\partial v_m}. \tag{S18}$$

Substituting Eq.(S18) into (S17), we solve ρ_V to be,

$$\rho_V = \frac{-(C\frac{\phi_m}{\tau_m} + \frac{\partial F}{\partial V}) \pm \sqrt{(C\frac{\phi_m}{\tau_m} + \frac{\partial F}{\partial V})^2 - 4C\frac{\phi_m}{\tau_m}(\frac{\partial F}{\partial V} + \frac{\partial F}{\partial v_m})}}{-2(\frac{\partial F}{\partial V} + \frac{\partial F}{\partial v_m})} \tag{S19}$$

Following the same procedure, we solve the situation for $H_2(\delta_{v_h}, \delta_{v_n}, \delta_{v_p}, \delta_{v_m}, \delta_V, \lambda_3, \lambda_4)$, and obtain the same result.

Further imposing the constraints $\rho_V, \rho_m \geq 0$ and utilizing the condition $\rho_V + \rho_m = 1$, we obtain,

$$\rho_V = \frac{-(C\frac{\phi_m}{\tau_m} + \frac{\partial F}{\partial V}) + \sqrt{(C\frac{\phi_m}{\tau_m} + \frac{\partial F}{\partial V})^2 - 4C\frac{\phi_m}{\tau_m}(\frac{\partial F}{\partial V} + \frac{\partial F}{\partial v_m})}}{-2(\frac{\partial F}{\partial V} + \frac{\partial F}{\partial v_m})}, \tag{S20}$$

$$\rho_m = 1 - \rho_V. \tag{S21}$$

2 THE DETAILS OF THE REDUCED MODEL

We summarize the final version of the reduced model,

$$\begin{aligned}\frac{C}{\rho_V}\frac{dV}{dt} =& -\underbrace{g_{Na}m_\infty^3(V)h_\infty(y)(V-E_{Na})}_{I_{Na}}-\underbrace{g_K n_\infty^4(y)(V-E_K)}_{I_K}\\ &-\underbrace{g_T p_\infty^2(y)q_\infty(z)(V-E_T)}_{I_T}-I_L-I_{KL}+\frac{10^{-3}}{A}I_{syn}(t),\end{aligned} \tag{S22a}$$

$$\frac{dy}{dt} =\phi_h\rho_h\frac{h_\infty(V)-h_\infty(y)}{\tau_h(V)h'_\infty(y)}+\phi_n\rho_n\frac{n_\infty(V)-n_\infty(y)}{\tau_n(V)n'_\infty(y)}+\phi_p\rho_p\frac{p_\infty(V)-p_\infty(y)}{\tau_p(V)p'_\infty(y)}, \tag{S22b}$$

$$\frac{dz}{dt} =\phi_q\frac{q_\infty(V)-q_\infty(z)}{\tau_q(V)q'_\infty(z)}. \tag{S22c}$$

Here, for convenience of description, we replace the new variable x with conventional notation of the membrane potential V. I_{syn} is the received synaptic current which is normalized by the membrane area $A = 1.43\times10^{-4}\mathrm{cm}^2$, I_{Na}, I_K and I_T are the currents mediated by the sodium, potassium and low-threshold calcium channels respectively, I_L is the leakage current, I_{KL} the potassium leaky current controlled by neuromodulators like acetylcholine and norepinephrine, $C = 1\,\mu\mathrm{F/cm}^2$ is the membrane capacitance, ρ_V is the reduction coefficient of the membrane potential V, y and z are two reduced equivalent potentials, ρ_h, ρ_n and ρ_p are reduction coefficients, $\phi_h = 1, \phi_n = 1, \phi_p = 6.9$ and $\phi_q = 3.7$ are temperature factors, $\tau_x(x \in \{h, n, p, q\})$ are time constant functions of each variable, and $x_\infty(x \in \{h, n, p, q\})$ are steady state functions.

Specifically, the reduction coefficients are expressed as:

$$\rho_p = k, \tag{S23a}$$

$$\rho_h = \frac{(1-k)\frac{\partial F}{\partial v_h}}{\frac{\partial F}{\partial v_h}+\frac{\partial F}{\partial v_n}}, \tag{S23b}$$

$$\rho_n = \frac{(1-k)\frac{\partial F}{\partial v_n}}{\frac{\partial F}{\partial v_h}+\frac{\partial F}{\partial v_n}}, \tag{S23c}$$

$$\rho_V = \frac{-(C\frac{\phi_m}{\tau_m}+\frac{\partial F}{\partial V})+\sqrt{(C\frac{\phi_m}{\tau_m}+\frac{\partial F}{\partial V})^2-4C\frac{\phi_m}{\tau_m}(\frac{\partial F}{\partial V}+\frac{\partial F}{\partial v_m})}}{-2(\frac{\partial F}{\partial V}+\frac{\partial F}{\partial v_m})}, \tag{S23d}$$

with

$$\partial F/\partial V = g_{Na}m_\infty^3(V)h_\infty(y)+g_K n_\infty^4(y)+g_T p_\infty^2(y)q_\infty(z)+g_L+g_{KL}, \tag{S24a}$$

$$\partial F/\partial v_m = g_{Na}h_\infty(y)(V-E_{Na})\cdot 3m_\infty^2(V)\cdot m'_\infty(V), \tag{S24b}$$

$$\partial F/\partial v_h = g_{Na}m_\infty^3(V)(V-E_{Na})\cdot h'_\infty(y), \tag{S24c}$$

$$\partial F/\partial v_n = g_K(V-E_K)\cdot 4n_\infty^3(y)\cdot n'_\infty(y). \tag{S24d}$$

(S24e)

For the sodium current I_{Na} (in the below, χ denotes membrane potential or equivalent potentials),

$$
\begin{aligned}
m_\infty(\chi) &= \frac{\alpha_m(\chi)}{\alpha_m(\chi)+\beta_m(\chi)}, & \tau_m(\chi) &= \frac{1}{[\alpha_m(\chi)+\beta_m(\chi)]},\\
h_\infty(\chi) &= \frac{\alpha_h(\chi)}{\alpha_h(\chi)+\beta_h(\chi)}, & \tau_h(\chi) &= \frac{1}{[\alpha_h(\chi)+\beta_h(\chi)]},\\
\alpha_m(\chi) &= \frac{0.32(13.-\chi+V_{th}^{Na})}{\exp\left[(13.-\chi+V_{th}^{Na})/4\right]-1}, & \beta_m(\chi) &= \frac{0.28(\chi-40.-V_{th}^{Na})}{\exp\left[(\chi-40.-V_{th}^{Na})/5\right]-1},\\
\alpha_h(\chi) &= 0.128\exp\left[(17-\chi+V_{th}^{Na})/18\right], & \beta_h(\chi) &= \frac{4}{1+\exp\left[(40-\chi+V_{th}^{Na})/5\right]},
\end{aligned}
$$

with the maximal conductance $g_{Na} = 100\,\mathrm{mS/cm^2}$ and the reversal potential $E_{Na} = 50\,\mathrm{mV}$, and the spike adjusting threshold $V_{th}^{Na} = -55\,\mathrm{mV}$. The derivatives $m'_\infty(\chi)$ and $h'_\infty(\chi)$ are given by

$$
m'_\infty(\chi) = \left[\alpha'_m(\chi)\beta_m(\chi) - \alpha_m(\chi)\beta'_m(\chi)\right] / \left[\alpha_m(\chi)+\beta_m(\chi)\right]^2 \tag{S25}
$$

$$
h'_\infty(\chi) = \left[\alpha'_h(\chi)\beta_h(\chi) - \alpha_h(\chi)\beta'_h(\chi)\right] / \left[\alpha_h(\chi)+\beta_h(\chi)\right]^2, \tag{S26}
$$

with

$$
\alpha'_m(\chi) = \frac{-0.32\left[\exp\left(\frac{13.-\chi+V_{th}^{Na}}{4}\right)-1\right] + 0.08(13.-\chi+V_{th}^{Na})\exp\left(\frac{13.-\chi+V_{th}^{Na}}{4}\right)}{\left[\exp\left(\frac{13.-\chi+V_{th}^{Na}}{4}\right)-1\right]^2} \tag{S27}
$$

$$
\beta'_m(\chi) = \frac{0.28\left[\exp\left(\frac{\chi-40.-V_{th}^{Na}}{5}\right)-1\right] - 0.056(\chi-40.-V_{th}^{Na})\exp\left(\frac{\chi-40.-V_{th}^{Na}}{5}\right)}{\left[\exp\left(\frac{\chi-40.-V_{th}^{Na}}{5}\right)-1\right]^2} \tag{S28}
$$

$$
\alpha'_h(\chi) = -0.128\exp\left[\frac{17-\chi+V_{th}^{Na}}{18}\right]/18 \tag{S29}
$$

$$
\beta'_h(\chi) = \frac{0.8\exp\left[\frac{40-\chi+V_{th}^{Na}}{5}\right]}{\left[1+\exp\left(\frac{40-\chi+V_{th}^{Na}}{5}\right)\right]^2}. \tag{S30}
$$

For the potassium current I_K,

$$
\begin{aligned}
n_\infty(\chi) &= \frac{\alpha(\chi)}{\alpha(\chi)+\beta(\chi)}, & \tau_n(\chi) &= \frac{1}{[\alpha_n(\chi)+\beta_n(\chi)]},\\
\alpha_n(\chi) &= \frac{0.032(15-\chi+V_{th}^{K})}{\exp\left[(15-\chi+V_{th}^{K})/5\right]-1}, & \beta_n(\chi) &= 0.5\exp\left[(10-\chi+V_{th}^{K})/40\right],
\end{aligned}
$$

where $V_{th}^{K} = -55\,\mathrm{mV}$, $E_K = -100\,\mathrm{mV}$, $g_K = 10\,\mathrm{mS/cm^2}$. The derivative of n_∞ is given by

$$n'_\infty(\chi) = \left[\alpha'_n(\chi)\beta_n(\chi) - \alpha_n(\chi)\beta'_n(\chi)\right] / \left[\alpha_n(\chi) + \beta_n(\chi)\right]^2, \tag{S31}$$

with

$$\alpha'_n(\chi) = \frac{-0.032\left[\exp(\frac{15-\chi+V_{th}^K}{5}) - 1\right] + 0.0064(15-\chi+V_{th}^K)\exp\left[\frac{15-\chi+V_{th}^K}{5}\right]}{\left[\exp(\frac{15-\chi+V_{th}^K}{5}) - 1\right]^2} \tag{S32}$$

$$\beta'_n(\chi) = -\frac{0.5}{40}\exp\left[(10-\chi+V_{th}^K)/40\right]. \tag{S33}$$

For the low-threshold calcium current I_T,

$$p_\infty(\chi) = \frac{1}{1+\exp\left[(-52-\chi+V_{th}^T)/7.4\right]}, \tag{S34}$$

$$q_\infty(\chi) = \frac{1}{1+\exp\left[(-80-\chi+V_{th}^T)/-5\right]}, \tag{S35}$$

$$\tau_p(\chi) = 3 + \frac{1}{\exp\left[(\chi+27-V_{th}^T)/10\right] + \exp\left[-(\chi+102-V_{th}^T)/15\right]}, \tag{S36}$$

$$\tau_q(\chi) = 85 + \frac{1}{\exp\left[(\chi+48-V_{th}^T)/4\right] + \exp\left[-(\chi+407-V_{th}^T)/50\right]}, \tag{S37}$$

where $g_T = 2.25\,\mathrm{mS/cm^2}$, $V_{th}^T = -3\,\mathrm{mV}$. The derivatives of $p_\infty(\chi)$ and $q_\infty(\chi)$ are given by

$$p'_\infty(\chi) = \frac{\exp\left[(-52-\chi+V_{th}^T)/7.4\right]}{7.4\left[1+\exp\left[(-52-\chi+V_{th}^T)/7.4\right]\right]^2}, \tag{S38}$$

$$q'_\infty(\chi) = \frac{\exp\left[(80+\chi-V_{th}^T)/5\right]}{-5\left[1+\exp\left[(80+\chi-V_{th}^T)/5\right]\right]^2}. \tag{S39}$$

The leakage current I_L is modeled as $I_L = g_L(V - E_L)$, with the leakage channel conductance $g_L = 0.06\,\mathrm{mS/cm^2}$ and the leakage reversal potential $E_L = -70\,\mathrm{mV}$.

The potassium leaky current I_{KL} is described by $I_{KL} = g_{KL}(V - E_{KL})$, with the potassium leakage channel conductance $g_{KL} = 0.0065\,\mathrm{mS/cm^2}$ and the reversal potential $E_{KL} = -100\,\mathrm{mV}$.

3 CURRENT-VOLTAGE RELATIONS

In this section, we will give the expression of current-voltage (I-V) relation in the reduced model by treating z as a parameter. According to Eq. (S22a), we can define the current I flows into the TRN cell when the

membrane potential is clamped to V as

$$\begin{aligned} I(V,y,z) &= \left[-I_{Na} - I_K - I_T - I_L - I_{KL} + 10^{-3} I_{syn}(t)/A\right]/\rho_V \\ &= \left[-g_{Na} m_\infty^3(V) h_\infty(y)(V - E_{Na}) - g_K n_\infty^4(y)(V - E_K) \right. \\ &\quad \left. - g_T p_\infty^2(y) q_\infty(z)(V - E_T) - I_L - I_{KL} + 10^{-3} I_{syn}(t)/A\right]/\rho_V. \end{aligned} \tag{S40}$$

By solving $dy/dt = 0$ in Eq. (S22b) we get $y(t) = V(t)$. Substituting it into Eq. (S22a), we get the I-V relation $I(V,z)$ under the given value of z as follows

$$\begin{aligned} I(V,z) = &\left[-g_{Na} m_\infty^3(V) h_\infty(V)(V - E_{Na}) - g_K n_\infty^4(V)(V - E_K) \right. \\ &\left. - g_T p_\infty^2(V) q_\infty(z)(V - E_T) - I_L - I_{KL} + 10^{-3} I_{syn}(t)/A\right]/\rho_V. \end{aligned} \tag{S41}$$

4 LINEAR STABILITY ANALYSIS

The system in Eq. (S22) can be rewritten as:

$$\frac{d\mathbf{x}}{dt} = \mathbf{F}(\mathbf{x}) \Longleftrightarrow \begin{cases} \frac{dx}{dt} = F_1(V,y,z) \\ \frac{dy}{dt} = F_2(V,y) \\ \frac{dz}{dt} = F_3(V,z) \end{cases} \tag{S42}$$

By solving $\frac{d\mathbf{x}}{dt} = \mathbf{0}$, we can solve the fixed point $\mathbf{x}^*$ which satisfies $\mathbf{F}(\mathbf{x}^*) = \mathbf{0}$.

The Jacobian matrix $\mathbf{J}(\mathbf{x})$ of the system at the fixed point $\mathbf{x}^*$ is given by

$$\mathbf{J}(\mathbf{x}^*) = \left.\begin{bmatrix} \frac{\partial F_1}{\partial V} & \frac{\partial F_1}{\partial y} & \frac{\partial F_1}{\partial z} \\ \frac{\partial F_2}{\partial V} & \frac{\partial F_2}{\partial y} & 0 \\ \frac{\partial F_3}{\partial V} & 0 & \frac{\partial F_3}{\partial z} \end{bmatrix}\right|_{V=V^*, y=y^*, z=z^*}. \tag{S43}$$

According to the dynamical system theory (Guckenheimer and Holmes, 2013), the stability of the fixed point can be evaluated by eigenvalues $(\lambda_1, \lambda_2, \lambda_3)$ of the Jacobian matrix $\mathbf{J}(\mathbf{x}^*)$. Specifically, there are two situations.

The fist condition is that all eigenvalues are real. Once all eigenvalues are positive, the fixed point is an **unstable node**. If eigenvalues are all negative, the fixed point is a **stable node**. When there is only one eigenvalue positive, the fixed point becomes a **saddle node**. The fixed point is classified to be an **unstable saddle** once there are two positive eigenvalues.

The second situation is that two eigenvalues are a pair of conjugate complex numbers (λ_1 and λ_2) and the third eigenvalue is a real number (λ_3). If $\lambda_3 > 0$ and **Re**$(\lambda_1) < 0$, the fixed point is an **unstable focus**. On the contrary, once $\lambda_3 < 0$ and **Re**$(\lambda_1) < 0$, the fixed point is a **stable focus**.

REFERENCES

Guckenheimer, J. and Holmes, P. (2013). *Nonlinear oscillations, dynamical systems, and bifurcations of vector fields*, vol. 42 (Springer Science & Business Media)

Kepler, T. B., Abbott, L., and Marder, E. (1992). Reduction of conductance-based neuron models. *Biological cybernetics* 66, 381–387

Maeda, Y., Pakdaman, K., Nomura, T., Doi, S., and Sato, S. (1998). Reduction of a model for an onchidium pacemaker neuron. *Biological cybernetics* 78, 265–276
